# The Repressive Effect of miR-148a on TGF beta-SMADs Signal Pathway Is Involved in the Glabridin-Induced Inhibition of the Cancer Stem Cells-Like Properties in Hepatocellular Carcinoma Cells

**DOI:** 10.1371/journal.pone.0096698

**Published:** 2014-05-07

**Authors:** Fei Jiang, Juan Mu, Xingxing Wang, Xianqing Ye, Lu Si, Shilong Ning, Zhong Li, Yuan Li

**Affiliations:** Department of Nutrition and Food Hygiene, The Key Laboratory of Modern Toxicology, Ministry of Education, School of Public Health, Nanjing Medical University, Nanjing, Jiangsu, People's Republic of China; Xiangya Hospital of Central South University, China

## Abstract

Hepatocellular carcinoma (HCC) is the third leading cause of cancer-related mortality worldwide. Current standard practices for treatment of HCC are less than satisfactory because of cancer stem cells (CSCs)-mediated post-surgical recurrence. For this reason, targeting the CSCs or the cancer cells with CSCs-like properties has become a new approach for the treatment of HCC. GLA exhibits anti-tumor effects in that it attenuates the proliferation, migration, invasion, and angiogenesis of human cancer cells. However, the functions of GLA in the regulation of CSCs-like properties in HCC cells, and the molecular mechanisms underlying in remain obscure. Here we found that GLA attenuated the CSCs-like properties by the microRNA-148a (miR-148a)-mediated inhibition of transforming growth factor beta (TGF-β)/SMAD2 signal pathway in HCC cell lines (HepG2, Huh-7, and MHCC97H). Indeed, GLA inhibited the activations/expressions of both TGFβ-induced and the endogenous SMAD2. Further, GLA improved the expression of miR-148a in a dose/time-dependent manner. MiR-148a, which targeted the *SMAD2*-3′UTR, decreased the expression and function of SMAD2. Knockdown of miR-148a abolished the GLA-induced inhibition of TGF-β/SMAD2 signal pathway and the CSCs-like properties in HCC cells. Our study found a novel mechanism that GLA inhibits the CSCs-like properties of HCC cells by miR-148a-mediated inhibition of TGF-β/SMAD2 signal pathway, which may help to identify potential targets for the therapies of HCC.

## Introduction

Hepatocellular carcinoma (HCC) is the most common liver malignancy and the third leading cause of cancer-related mortality worldwide [1]. Current standard practices for treatment of HCC, surgical resection and chemotherapy are less than satisfactory because of metastasis and post-surgical recurrence [1]. A concept proposed to explain the characteristics of neoplastic tissues is the existence of self-renewing, stem-like cells, called cancer stem cells (CSCs) [2]. CSCs have been identified in various human cancers, including HCC. Within a tumor, CSCs, which constitute a small portion of the neoplastic cells, are defined by their capacity to produce new tumors [3]. For this reason, targeting the cancer cells with CSCs-like properties has become the new way for the treatment of human liver cancers.

The root of *glycyrrhiza glabra* (licorice) has been used for many centuries in Asia and Europe as an antioxidant, antidote, demulcent, expectorant and a remedy for allergic inflammation, as well as a flavoring and sweetening agent [4]. Glabridin [GLA, (*R*)-4-(3, 4-dihydro-8, 8-dimethyl)-2*H*, 8*H*-benzo [1, 2-*b*: 3, 4-*b*'] dipyran-3yl)-1, 3-benzenediol] is a polyphenolic flavonoid and a main constituent in the hydrophobic fraction of licorice extract [5]. In addition to estrogenic effects, GLA exhibits a wide range of biological activities including neuro-protective, cardiovascular-protective, anti-inflammatory, etc [5]–[7]. Recent studies indicate that GLA presents anti-tumor effects, with attenuation of the proliferation, migration/invasion, and angiogenesis in human cancer cells [8], [9]; However, the effects of GLA on the CSCs-like properties in HCC cells, and the molecular mechanisms underlying in remain obscure.

Transforming growth factor beta (TGF-β) is a critical regulator involved in the cell growth, differentiation, and development [10]. It is also the most potent hepatic pro-fibrogenic cytokine predominantly produced by activated mesenchymal cells upon chronic liver damage [11]. In the initiation and development of various tumors, TGF-β induces the epithelial-mesenchymal transition (EMT), which is a critical cellular event in the acquirement of CSCs-like properties [12], [13]. Therefore, TGF-β inhibitors have been developed for anti-cancer therapies [14], [15]. Current study indicates that inhibition of TGF-β blocks the generation of CSCs, which enhances the chemotherapy action against triple-negative breast cancer [16]. In the present study, we treated HCC cell lines (HepG2, Huh-7, and MHCC97H) by GLA to determine the early molecular changes, with emphases on CSCs-like properties and TGF-β pathway.

## Materials and Methods

### Cell culture and reagents

HCC cell lines (HepG2 and Huh-7) and Human normal liver cell line (L-02) were obtained from the Shanghai Institute of Cell Biology, Chinese Academy of Sciences (Shanghai, China). MHCC97H cell line (HCC cells with a high migratory potential) was obtained from Liver Cancer Institute, Zhongshan Hospital, Fudan University, Shanghai, China. Cells were maintained in 5% CO_2_ at 37 °C in Dulbecco's Modified Eagle Medium (DMEM, Life Technologies/Gibco, Grand Island, NY) supplemented with 10% fetal bovine serum (FBS, Life Technologies/Gibco), 100 U/ml penicillin, and 100 µg/ml streptomycin (Life Technologies/Gibco, Gaithersburg, MD). GLA (≥98.0% purity) was purchased from Sigma Chemical Co. (St. Louis, MO, USA). All other reagents used were of analytical grade or the highest grade available.

### Reverse-transcriptase polymerase chain reaction (RT-PCR)

Total RNA (2 µg) was transcribed into cDNA using AMV Reverse Transcriptase (Promega, Madison, USA). Primers used are as listed in Table S1. The PCR reaction was evaluated by checking the PCR products on 2% w/v agarose gels. Bands were normalized by use of *Glyceraldehyde 3-phosphate dehydrogenase (GAPDH)* to correct for differences in loading of the cDNAs.

### Quantitative real-time polymerase chain reaction (qRT-PCR)

Total RNA (1 µg) was transcribed into cDNA using the TaqMan miRNA Reverse Transcription Kit (Applied Biosystems, Foster City, CA) with miRNA-specific looped reverse primers. The reaction conditions were as follows: 42 °C for 15 min and 85 °C for 5 s. qRT-PCR was conducted using a TaqMan PCR kit by Applied Biosystems 7300 Sequence Detection System (Applied Biosystems) for 40 cycles of 95 °C for 15 s and 60 °C for 1 min. The *U6* snRNA was used as an internal control. Fold changes in expression of each gene were calculated by a comparative threshold cycle (Ct) method using the formula 2^−(ΔΔCt)^.

### Western blots

Cell lysates were separated by sodium dodecyl sulfate (SDS)-polyacrylamide gel electrophoresis and transferred to polyvinylidene fluoride (PVDF) membranes (Millipore, Billerica, USA); the immune complexes were detected by enhanced chemiluminescence (Cell Signaling Technology, Beverly, MA, USA). Antibodies used were SMAD2 and p-SMAD2 (Ser 465/467, Cell Signaling Technology); GAPDH (Sigma). Blots were normalized by use of GAPDH to correct for differences in loading of the proteins.

### Spheroid formation

In non-adherent 24-wells dishes (Costar, US), treated cells (2×10^3^) were suspended in defined, serum-free medium composed of DMEM/F-12 (Gibco), 10 ng/ml of human recombinant basic fibroblast growth factor (bFGF, R&D Systems, USA), and 10 ng/ml of epidermal growth factor (EGF, R&D Systems). Cells were grown for 10 days, and then counted under a microscope (Olympus, Tokyo, Japan).

### Anchorage-independent growth

Soft agar plates were prepared in 24-wells dishes with under-layers of 0.70% agarose in DMEM medium supplemented with 10% FBS. To test their capacity for colony growth in soft agar, cells were plated in triplicate at a density of 1×10^3^ in 2 mL of 0.35% agarose over the agar base. Cultures were fed every three days; after for 14 days, colonies were counted under a microscope (Olympus).

### Cell transfection

Anti-con, anti-miR-148a, Con-mimic, and miR-148a-mimic were synthesized by RiBoBio Co. Cells were transiently transfected using the Lipofectamine 2000 reagent (Invitrogen, Carlsbad, USA) for 12 h, according to the manufacturer's protocol. For gene recovery assay, after MHCC97H cells were transfected by anti-miR-148a for 12 h, they were cultured in fresh DMEM medium supplemented with 10% FBS (Gibco), 100 U/ml penicillin, and 100 µg/ml streptomycin (Gibco) for another 24 h, followed by transfected with Con-mimic or miR-148a-mimic for 12 h.

### Luciferase reporter assay

The pGL3-*SMAD2*-3′UTR-Luc construct was purchased from Shuntian Biology (Shanghai). The plasmid phRL-tk (used as internal control for transfection efficiency and cytotoxicity of test chemicals) containing the Renilla luciferase gene was purchased from Promega. Briefly, HepG2 cells were plated in 24-wells cell culture dishes. The cells proliferated to 60 to 80% confluence after 24 h of culture. Anti-con or anti-miR-148a was co-transfected with the reporter constructs respectively, by using Lipofecamine 2000 reagent (Invitrogen) according to the manufacturer's protocol. After an incubation period of 12 h, the transfection medium was replaced. Then, after the cells were treated by 0 or 20 µM of GLA for 24 h, they were harvested, washed with PBS (pH 7.4). The cells were lysed with passive lysis buffer (Promega). The cell lysates were analyzed immediately with a 96-well plate luminometer (Berthold Detection System, Pforzheim, Germany). The amounts of luciferase and Renilla luciferase were measured with the Dual-Luciferase Reporter Assay System Kit (Promega) following the manufacturer's instructions. The values of luciferase activity for each lysate were normalized to the Renilla luciferase activity. The relative activity was converted into fold induction above the vehicle control value.

### Statistical analysis

Derived values were presented as the means ± SD. A Student's *t* test, and an one-way analysis of variance (ANOVA) followed by Dunnett's *t* test were used to assess significant differences between groups. *P* values <0.05 were considered statistically significant.

## Results

### GLA attenuates the CSCs-like properties in HCC cells

To determine the concentrations of GLA using in our study, we exposed HepG2 or L-02 cells to 0, 5, 10, 20, 40, or 80 µM of GLA for 24, 48, or 72 h. As shown in Figures. S1A and S1B, there was no detectable effect of 10 or 20 µM GLA on cell viabilities neither in HCC cells (HepG2) nor in normal liver cells (L-02). So we chose these concentrations for further investigation.

**Figure 1:**
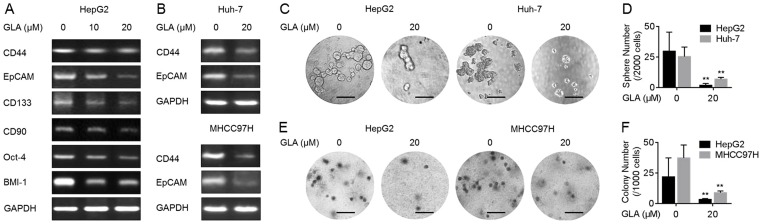
GLA attenuates the CSCs-like properties in HCC cells. (**A**) HepG2 cells were treated by 0, 10, or 20 µM GLA for 72 h. RT-PCR analyses of *CD44*, *EpCAM*, *CD133*, *CD90*, *Oct-4*, and *BMI-1*; (**B**) Huh-7 and MHCC97H cells were treated by 0 or 20 µM GLA for 72 h. RT-PCR analyses of *CD44* and *EpCAM*; (C and D) HepG2 and Huh-7 cells were treated by 0 or 20 µM GLA for 72 h. (**C**) Free-floating, viable spheres formed by cells (bar = 250 µm); (**D**) Sphere quantitation (mean ± SD, n = 3); (E and F) HepG2 and MHCC97H cells were treated by 0 or 20 µM GLA for 72 h. (**E**) Colony formation in the soft agar (bar  = 250 µm); (**F**) Colony numbers (mean ± SD, n = 3). **p<0.01 compared with medium control cells (student's *t* test).

An increased exhibition of CSCs-like properties plays a key role in the initiation, development, and outcome of diverse cancer, including HCC [2]. *CD44*, *CD90*, *CD133*, and *EpCAM* are the cell–surface markers of liver cancer stem cells [17]–[19], further, *Oct-4* and *BMI-1* are the key ‘stemness’ genes in CSCs from various cancers [13], [20]. Here, as shown in Figure. 1A and 1B, GLA decreased the expressions of these genes in HepG2 cells in a dose-dependent manner; meanwhile, the decreased expressions of *CD44* and *EpCAM* were also observed in another two HCC cells (Huh-7 and MHCC97H).

Formation of spheroids demonstrates the capacity of cells for self-renewal and for the initiation/development of tumors [2]. Then, the capacity of HCC cells for the formation of spheroids during GLA treatment was determined. As shown in Figures. 1C and 1D, GLA decreased the formation of spheroids in HepG2 and Huh-7 cells. Anchorage-independent growth is a characteristic of malignant cells [21]. We further determined the effects of GLA on the malignant properties in HCC cells. As shown in Figures. 1E and 1F, GLA decreased the anchorage-independent growth in HepG2 and MHCC97H cells.

### TGF-β/SMADs signal pathway is involved in the elevation of CSCs-like properties in HepG2 cells

**Figure 2:**
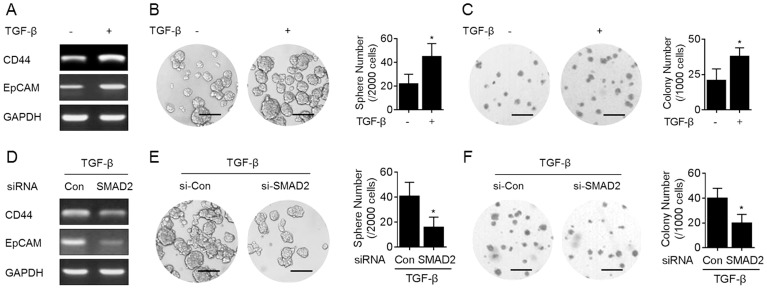
TGF-β/SMADs signal pathway is involved in the elevation of CSCs-like properties in HepG2 cells. (A–C) HepG2 cells were treated by 0 or 10 ng/ml TGF-β for 48 h. (D–F) After HepG2 cells were transfected by 10 nM SMAD2-siRNA for 12 h, they were treated by 10 ng/ml TGF-β for 48 h. (**A and D**) RT-PCR analyses of *CD44* and *EpCAM*; (**B and E**, left) Free-floating, viable spheres formed by cells (bar  = 250 µm); (**B and E**, right) Sphere quantitation (mean ± SD, n = 3); (**C and F**, left) Colony formation in the soft agar (bar  = 250 µm); (**C and F**, right) Colony numbers (mean ± SD, n = 3). *p<0.05 compared with medium control cells or Con-siRNA-transfected cells treated by TGF-β (student's *t* test).

TGF-β/SMADs pathway has been shown to increase the stem-like properties in human cancer cells [22]. Here we found that TGF-β improved the expressions of CD44 and EpCAM (Figure. 2A). Further, in TGF-β-treated HepG2 cells, the abilities of spheroids formation and anchorage-independent growth were enhanced (Figures. 2B and 2C). However, in SMAD2 (a classical down-stream factor regulated by TGF-β, [16]) knockdown HepG2 cells, such phenomenon induced by TGF-β was attenuated (Figures. 2D-2F).

### GLA blocks the TGF-β/SMAD2 signal pathway in HCC cells

**Figure 3:**
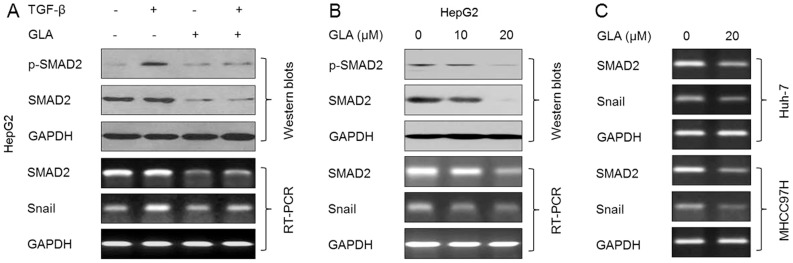
GLA blocks the TGF-β/SMAD2 signal pathway in HCC cells. (**A**) After HepG2 cells were pre-treated by 0 or 20 µM GLA for 12 h, they were exposed to 0 or 10 ng/ml TGF-β for 24 h. (top) Western blot analyses of p-SMAD2 and SMAD2, (bottom) RT-PCR analyses of *SMAD2* and *snail*; (**B**) HepG2 cells were treated by 0, 10, or 20 µM GLA for 72 h. (top) Western blots analyses of p-SMAD2 and SMAD2, (bottom) RT-PCR analyses of *SMAD2* and *snail*; (**C**) Huh-7 and MHCC97H cells were treated by 0 or 20 µM GLA for 72 h. RT-PCR analyses of *SMAD2* and *snail*.

We then examined the effects of GLA on the TGF-β-induced activation of SMAD2. As shown in Figure. 3A, GLA blocked the TGFβ-induced phosphorylation of SMAD2 and the expression of *Snail* (the downstream gene of SMAD2, [23]); interestingly, GLA also attenuated the expression of total SMAD2 mRNA and protein in the presence or absence of TGF-β. So we next determined the effects of GLA on the expressions of endogenous SMAD2. As shown in Figures. 3B and 3C, GLA decreased the expression and activation of endogenous SMAD2 in HCC cells (HepG2, Huh-7, and MHCC97H). As the fact that *SMAD2* mRNA was reduced by GLA, we hypothesized that this repressive effect might be mediated by the miRNAs.

### GLA improves the expression of miR-148a in HCC cells

**Figure 4:**
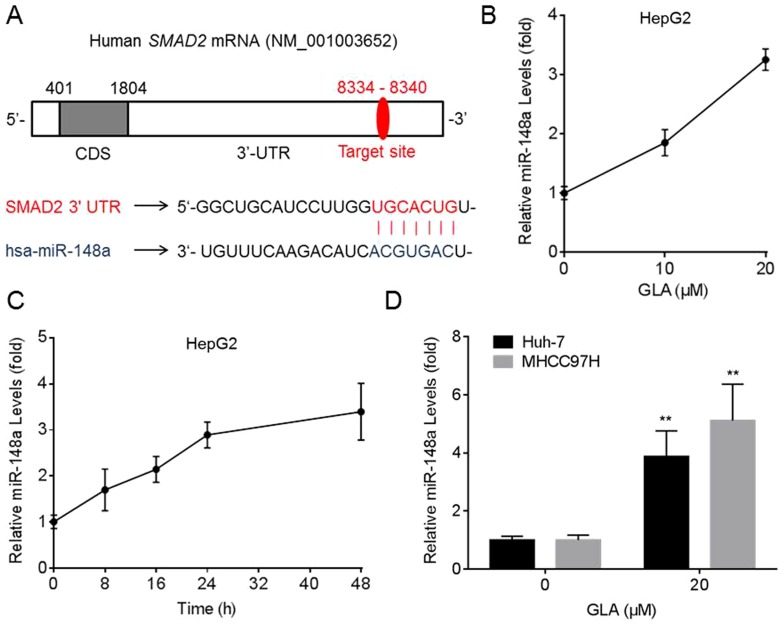
GLA improves the expression of miR-148a in HCC cells. (**A**) The target sequences of miR-148a in the 3′-UTR of *SMAD2*; (**B**) HepG2 cells were treated by 0, 10, or 20 µM GLA for 24 h. qRT-PCR analyses of the expression of miR-148a (mean ± SD, n = 3); (**C**) HepG2 cells were treated by 0 or 20 µM GLA for 0, 8, 16, 24, or 48 h. qRT-PCR analyses of the expression of miR-148a (mean ± SD, n = 3); (**D**) Huh-7 and MHCC97H cells were treated by 0 or 20 µM GLA for 24 h. qRT-PCR analyses of the expression of miR-148a (mean ± SD, n = 3). **p<0.01 compared with medium control cells (student's *t* test).

By using TargetScan 6.2 (www.targetscan.org), we found that miR-148a was predicted to bind the *SMAD2*-3′ UTR. The target sites of miR-148a in *SMAD2* mRNA were exhibited in Figure. 4A. We then determined the effects of GLA on the expression of miR-148a in HCC cells. As shown in Figures. 4B and 4C, GLA improved the expression of miR-148a in a dose/time-dependent manner in HepG2 cells. Meanwhile, GLA also elevated the expression of miR-148a in Huh-7 and MHCC97H cells (Figure 4D).

### GLA inhibits the SMAD2 by miR-148a in HCC cells

**Figure 5:**
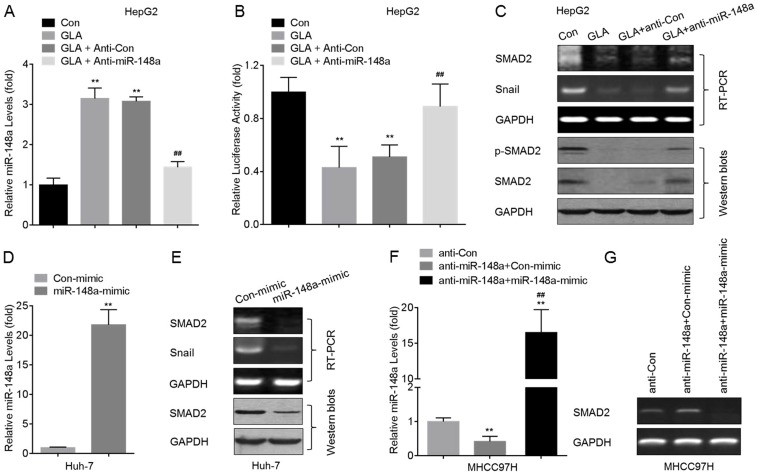
GLA inhibits the SMAD2 by miR-148a in HCC cells. (A–C) After HepG2 cells were pre-transfected by anti-con or anti-miR-148a for 12 h, they were exposed to 0 or 20 µM of GLA for 72 h. (**A**) qRT-PCR analyses of the expression of miR-148a (mean ± SD, n = 3); (**B**) Luciferase reporter assays analysis of the effects of miR-148a on *SMAD2* 3′UTR; (**C**) RT-PCR analyses of *SMAD2* and *snail* (top), and Western blots analyses of p-SMAD2 and SMAD2 (bottom). **p<0.01 compared with medium control cells, and ^##^p<0.01 compared with HepG2 cells treated by GLA alone or with anti-con-transfected HepG2 cells treated by GLA (ANOVA followed by Dunnett's *t* test). (D and E) Huh-7 cells were transfected by con-mimic or miR-148a-mimic for 12 h. (**D**) qRT-PCR analyses of the expression of miR-148a (mean ± SD, n = 3); (**E**) RT-PCR analyses of *SMAD2* and *snail* (top), and Western blots analyses of SMAD2 (bottom); **p<0.01 compared with Huh-7 cells transfected by con-mimic (student's t test). (F and G) After MHCC97H cells were transfected by anti-miR-148a for 12 h, they were cultured in fresh DMEM medium supplemented with 10% FBS, 100 U/ml penicillin, and 100 µg/ml streptomycin for another 24 h, followed by transfected with con-mimic or miR-148a-mimic for 12 h. (**F**) qRT-PCR analyses of the expression of miR-148a (mean ± SD, n = 3); (**G**) RT-PCR analyses of *SMAD2*. **p<0.01 compared with MHCC97H cells transfected by anti-con, ^##^p<0.01 compared with MHCC97H cells transfected by anti-miR-148a plus con-mimic (ANOVA followed by Dunnett's *t* test).

Based on the prediction that there are target sites of miR-148a in *SMAD2* mRNA and on that GLA elevated the expression of miR-148a, we hypothesized that miR-148a might be involved in the GLA-induced decreased expression of SMAD2. Here, knockdown of miR-148a (Figure. 5A) led to a significant increase of the luciferase activity (Figure. 5B), and blocked the GLA-induced decreased expression and activation of SMAD2 in HepG2 cells (Figure. 5C). Meanwhile, overexpression of miR-148a (Figure. 5D) in Huh-7 cells decreased the expression and activation of SMAD2 (Figure. 5E). Moreover, we used gene recovery assay to further confirm our conclusion. In MHCC97H cells, knockdown of miR-148a elevated the expression of *SMAD2*, however, restoration of miR-148a by mimic abolished such effect (Figure. 5F and 5G).

### GLA attenuates the CSCs-like properties in HCC cells by miR-148a

**Figure 6:**
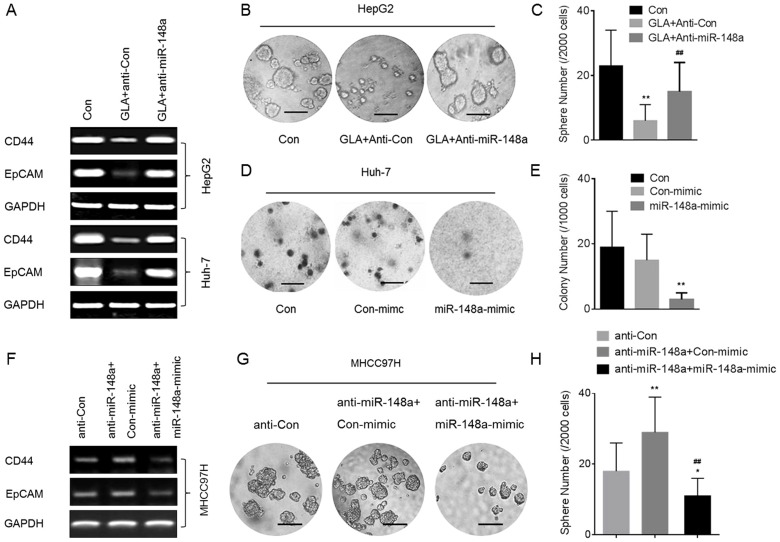
GLA attenuates the CSCs-like properties in HCC cells by miR-148a. (A-C) After HepG2 or Huh-7 cells were pre-transfected by anti-con or anti-miR-148a for 12 h, they were exposed to 0 or 20 µM GLA for 72 h. (**A**) RT-PCR analyses of *CD44* and *EpCAM*; (**B**) Free-floating, viable spheres formed by HepG2 cells (bar  = 250 µm); (**C**) Sphere quantitation (mean ± SD, n = 3); **p<0.01 compared with medium control HepG2 cells, and ^##^p<0.01 compared with anti-con-transfected HepG2 cells treated by GLA (ANOVA followed by Dunnett's *t* test). (D and E) Huh-7 cells were transfected by con-mimic or miR-148a-mimic for 12 h. (**D**) Colony formation in the soft agar (bar  = 250 µm); (**E**) Colony numbers (mean ± SD, n = 3). **p<0.01 compared with medium control Huh-7 cells or with Huh-7 cells transfected by con-mimic (ANOVA followed by Dunnett's *t* test). (F–H) MHCC97H cells were treated as described in Figure. 5F. (**F**) RT-PCR analyses of *CD44* and *EpCAM*; (**G**) Free-floating, viable spheres formed by MHCC97H cells (bar  = 250 µm); (**H**) Sphere quantitation (mean ± SD, n = 3); **p<0.01 compared with MHCC97H cells transfected by anti-con, ^##^p<0.01 compared with MHCC97H cells transfected by anti-miR-148a plus con-mimic (ANOVA followed by Dunnett's *t* test).

Since TGF-β/SMAD2 improves the CSCs-like properties, and since miR-148a targets SMAD2, we hypothesized that GLA attenuates the CSCs-like properties by miR-148a in HCC cells. Here, knockdown of miR-148a blocked the GLA-induced decreased expressions of *CD44* and *EpCAM* mRNA (Figure. 6A). For HepG2 cells, knockdown of miR-148a blocked the GLA-induced decreased formation of spheroids (Figures. 6B and 6C). For Huh-7 cells, overexpression of miR-148a attenuated the capacity of anchorage-independent growth (Figures. 6D and 6E). Then we used gene recovery assay to further confirm our conclusion. In MHCC97H cells, knockdown of miR-148a elevated the expression of *CD44* and *EpCAM*, and improved the formation of spheroids; however, restoration of miR-148a by mimic abolished such effect (Figure. 6F-6H).

## Discussions

Current chemotherapy against HCC usually targets the bulk population of tumor directly, which is able to shrink the primary tumor, however, it fails to consistently eradicate the lesions. The discovery of CSCs has changed our view of carcinogenesis and chemotherapy. CSCs, also been termed ‘tumor initiating cells’, have the capacity to produce new tumors. Based on this concept, CSCs are responsible for the formation and growth of neoplastic tissue and are resistant to chemotherapeutic agents, explaining why traditional drugs can initially shrink a tumor but fail to eradicate it, allowing recurrence [24]. Here, we chose the HepG2, Huh-7, and MHCC97H cell lines to study the effects of GLA on the CSCs-like properties because these cell lines exhibited CSCs-like properties, and have been used to investigate the effects of phytochemicals on the CSCs-like “side population” cells [24]–[27].

GLA, an isoflavonoid of *G. glabra L. roots*, inhibits the tyrosinase-dependent melanin biosynthesis effectively, suggesting that it may serve as a candidate for skin-lightening agents [5]. Besides, it has also been associated with a wide range of biological properties such as antioxidant, anti-inflammatory, estrogenic, neuroprotective, etc [5]–[7]. Recent studies reveal the anti-cancer effects induced by GLA, that it prevents the oxidative DNA fragmentation in UVB-irradiated human keratinocyte HaCaT cells [28]; meanwhile, it blocks the proliferation of human breast cancer cells [8]; moreover, it inhibits the migration, invasion, and angiogenesis by inhibiting the FAK/Rho signaling pathway [29]; further, it also enhances the efficacy of chemotherapy by inhibiting P-glycoprotein and multidrug resistance protein 1 synthesis [30]. Here we identified that GLA attenuated (**a**) the expressions of CD44, CD133, CD90, and EpCAM, (**b**) the capacity of spheroids formation (a marker for self-renewal), and (**c**) the capacity of anchorage-independent growth (a character of malignant cells) in HepG2, Huh-7, and MHCC97H cells, suggesting a novel function that GLA could regulate the CSCs-like properties in HCC cells.

In the liver, TGF-β is an important link among chronic injury, cirrhosis, and HCC, and may be served as a key target for HCC therapy [15], [31]. TGF-β signaling is initiated by the binding of TGF-β to TGF-β receptor II (TGFβ-RII), followed by the activation of TGFβ-RI, Smad2/3 phosphorylation, and formation of the Smad2/3/4 complexes [10]. There is a relationship between the TGF-β and CSCs, with the evidence suggesting that TGF-β induces EMT, which leads to the acquirement of CSCs-like properties [12], [13]. Here we found that (**a**) TGF-β treatment induced putative cancer stem markers and increased anchorage-independent growth and formation of spheroids in HepG2 cells and (**b**) knockdown of Smad2 reversed these effects, suggesting that TGF-β signaling played an important role in enhancing the CSCs-like properties in HCC cells. However, an association between the TGF-β and GLA-regulated CSCs-like properties has not been examined previously. In the present study, GLA decreased the TGF-β-induced phosphorylation of SMAD2. Importantly, GLA attenuated the expression and activation of endogenous SMAD2 in HCC cells, indicating that TGF-β/SMAD2 pathway might be involved in the GLA-induced repressive effect on the CSCs-like properties in HCC cells.

MiRNAs are the non-encoding small RNA oligonucleotides that regulate gene expression [32]. In several tumors some miRNAs are differentially expressed relative to normal tissues [33]. However, the miRNAs networks and their regulation of mRNA translation and protein expression in the CSCs-like properties in HCC cells remain to be elucidated. MiR-148a is a pro-apoptotic miRNA by targeting Bcl-2 [34]. In addition, inhibition of miR-148a by hyper-methylation is associated with metastasis in many tumor types and with up-regulation of metastasis-associated genes [35]. In the liver, miR-148a was first shown to modulate the levels of cytochrome P450 3A4 via post-transcriptionally regulating the 3′UTR of the Pregnane X Receptor (*PXR*) mRNA [36]. Previous study suggests that miR-148a is involved in the anti-metastasis of HCC cells by the inhibitions of Wnt1-mediated EMT and acquirement of CSCs-like properties [24]. Here, by using TargetScan 6.2, we found that miR-148a was predicted to bind the *SMAD2*-3′ UTR. Moreover, knockdown of miR-148a led to significant increases of the expression/activation of SMAD2 and CSCs-like properties in GLA-treated HepG2 cells. Further, overexpression of miR-148a decreased the expression/activation of SMAD2 and CSC-like properties in Huh-7 and MHCC97H cells. These results suggested that the inhibition of SMAD2 by miR-148a might mediate the GLA-attenuated CSCs-like properties in HCC cells.

**Figure 7:**
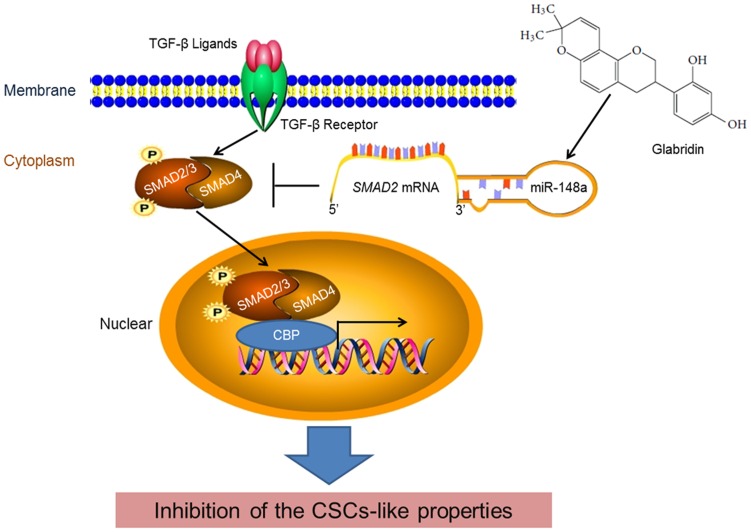
The repressive effect of miR-148a on TGF-β/SMADs signal pathway is involved in the GLA-induced inhibition of the CSCs-like properties in HCC cells.

In conclusion, GLA attenuated the CSCs-like properties by the inhibition of TGF-β/SMADs pathway. Indeed, GLA improved the expression of miR-148a, which targeted the SMAD2-3′UTR and down-regulated the SMAD2 expression/activation. Knockdown of miR-148a abolished the GLA-induced inhibition of TGF-β/SMAD2 and the CSCs-like properties in HCC cells (Figure. 7). Understanding a novel mechanism, by which GLA inhibits the CSCs-like properties of HCC cells, our study may help to identify potential targets for the therapies of HCC.

## Supporting Information

Figure S1 **Effects of GLA on the viability and CSCs markers in HepG2 and L-02 cells.** (**A and B**) HepG2 or L-02 cells were treated by 0, 5, 10, 20, 40, or 80 µM GLA for 24, 48, or 72 h, respectively. The cells viabilities were evaluated by WST-8 hydrolysis using a Cell Counting Kit-8 assay. The relative ratios of cell viability were determined by comparing of cells exposed to no GLA. (**C**) L-02 cells were treated by 0 or 20 µM GLA for 72 h. qRT-PCR analyses of the expression of *CD44*, *EpCAM*, and *CD133* (mean ± SD, n = 3).
(TIF)

Table S1 Primers used for RT-PCR.
(DOCX)
