# Supplementary figures and images for: The Repressive Effect of miR-148a on TGF beta-SMADs Signal Pathway Is Involved in the Glabridin-Induced Inhibition of the Cancer Stem Cells-Like Properties in Hepatocellular Carcinoma Cells
Source: PLoS One. 2014 May 7;9(5):e96698. doi: 10.1371/journal.pone.0096698 (PMC4013140; doi:10.1371/journal.pone.0096698)

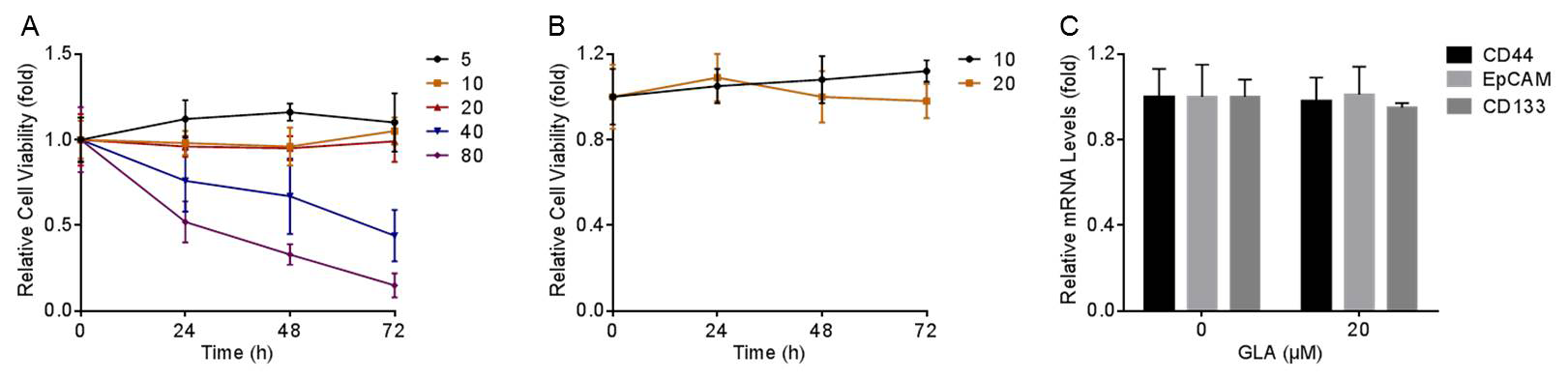

Supplement: Figure S1 — Effects of GLA on the viability and CSCs markers in HepG2 and L-02 cells. (A and B) HepG2 or L-02 cells were treated by 0, 5, 10, 20, 40, or 80 µM GLA for 24, 48, or 72 h, respectively. The cells viabilities were evaluated by WST-8 hydrolysis using a Cell Counting Kit-8 assay. The relative ratios of cell viability were determined by comparing of cells exposed to no GLA. (C) L-02 cells were treated by 0 or 20 µM GLA for 72 h. qRT-PCR analyses of the expression of CD44, EpCAM, and CD133 (mean ± SD, n = 3). (TIF) [file pone.0096698.s001.tif]
