# Supplementary material for: The Repressive Effect of miR-148a on TGF beta-SMADs Signal Pathway Is Involved in the Glabridin-Induced Inhibition of the Cancer Stem Cells-Like Properties in Hepatocellular Carcinoma Cells
Source: PLoS One. 2014 May 7;9(5):e96698. doi: 10.1371/journal.pone.0096698 (PMC4013140; doi:10.1371/journal.pone.0096698)
Supplement: Table S1 — Primers used for RT-PCR. (DOCX) [file pone.0096698.s002.docx]

**Table S1. Primers used for RT-PCR.**

| Genes | Primers (5’-3’) |
| --- | --- |
| CD44 | TGAGCATCGGATTTGAGAC (F)  CATACTGGGAGGTGTTGGA (R) |
| CD90 | AAGGAGAAACAGGAAACCTC (F)  ACAGACACAGTCCAACTTCC (R) |
| CD133 | GCACTCTATACCAAAGCGTCA (F)  CCATACTTCTTAGTTTCCTCA (R) |
| EpCAM | AAGGAGAAACAGGAAACCTC (F)  ACAGACACAGTCCAACTTCC (R) |
| Oct-4 | GCTTCCTCCACCCACTTCT (F)  GTATTCAGCCAAACGACCAT (R) |
| BMI-1 | CTGATGACCCATTTACTGA (F)  CTCCACCTCTTCTTGTTTG (R) |
| SMAD2 | GTTCCTGCCTTTGCTGAC (F)  TCTCTTTGCCAGGAATGCTT (R) |
| Snail | TTCTCCCGAATGTCCCT (F)  TCAGCCTTTGTCCTGTAGC (R) |
